# Linking the effect of localised pitting corrosion with mechanical integrity of a rare earth magnesium alloy for implant use

**DOI:** 10.1016/j.bioactmat.2022.08.004

**Published:** 2022-08-12

**Authors:** Kerstin van Gaalen, Conall Quinn, Felix Benn, Peter E. McHugh, Alexander Kopp, Ted J. Vaughan

**Affiliations:** a Biomechanics Research Centre (BioMEC), Biomedical Engineering, School of Engineering, National University of Ireland Galway, Galway, Ireland; b Meotec GmbH, Aachen, Germany; c School of Mechanical and Aerospace Engineering, Queen's University Belfast, Belfast, United Kingdom

**Keywords:** Magnesium, Localised corrosion, Corrosion modelling, Phenomenology, FEM

## Abstract

This study presents a computational framework that investigates the effect of localised surface-based corrosion on the mechanical performance of a magnesium-based alloy. A finite element-based phenomenological corrosion model was used to generate a wide range of corrosion profiles, with subsequent uniaxial tensile test simulations to predict the mechanical response to failure. The python-based detection framework *PitScan* provides detailed quantification of the spatial phenomenological features of corrosion, including a full geometric tracking of corroding surface. Through this approach, this study is the first to quantitatively demonstrate that a surface-based non-uniform corrosion model can capture both the geometrical and mechanical features of a magnesium alloy undergoing corrosion by comparing to experimental data. Using this verified corrosion modelling approach, a wide range of corrosion scenarios was evaluated and enabled quantitative relationships to be established between the mechanical integrity and key phenomenological corrosion features. In particular, we demonstrated that the minimal cross-sectional area parameter was the strongest predictor of the remaining mechanical strength (R^2^ = 0.98), with this relationship being independent of the severity or spatial features of localised surface corrosion. Interestingly, our analysis demonstrated that parameters described in ASTM G46-94 showed weaker correlations to the mechanical integrity of corroding specimens, compared to parameters determined by *Pitscan*. This study establishes new mechanistic insight into the performance of the magnesium-based materials undergoing corrosion.

## Highlights

• Corrosion profile (uniform/localised) generation with phenomenological degradation model.
• Automated phenomenological tracking of corrosion features by PitScan.
• Linking mechanical key parameters and geometrical corrosion features including in-vitro data.
• Identification of profile independent corrosion features and model fitting.

## Graphical abstract

**Figure:**
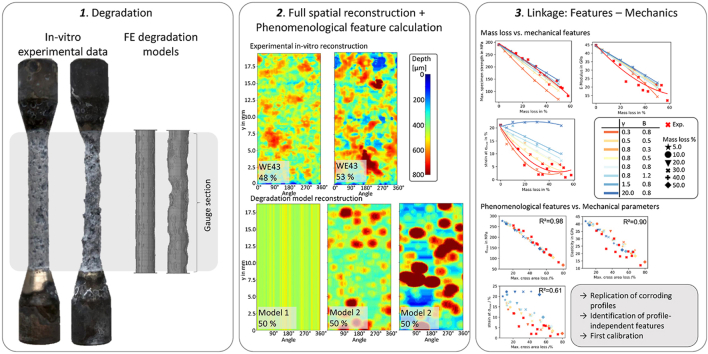

## 1 Introduction

Magnesium and its alloys have significant potential in orthopaedic applications as they have osteoconductive properties [[1], [2], [3], [4], [5], [6]] and have similar mechanical properties to native bone [7]. Magnesium-based alloys are also biodegradable, whereby the implant is gradually removed from the body once its load bearing function is completed. This reduces the need for revision surgeries, thereby reducing patient risk and cost to health systems. However, magnesium-based alloys can undergo increased and localised corrosion, which may lead to an unwanted early failure of the implant. In unloaded physiological scenarios, magnesium-based alloys degrade through several surface-based corrosion mechanisms including galvanic, inter-granular and pitting corrosion [8]. The localised corrosion mechanisms are generally caused by impurities and inhomogeneities in the material, which are largely unavoidable due to the manufacturing process of such alloys. While the spatial and temporal evolution of corrosion can be controlled to some degree by varying alloying composition and/or by applying a surface coating [[8], [9], [10], [11], [12]], it is generally not possible to achieve uniform, or non-localised, corrosion in magnesium-based biomaterials [[13], [14], [15], [16], [17]]. Despite this, many studies investigating the degradation performance of magnesium alloys only consider bulk measures of corrosion, evaluated by gravimetric methods, hydrogen evolution, μCT images, or electrochemical tests, and ignore aspects of localised surface corrosion, which can greatly impact overall performance and cause early failures of devices. While some studies provide limited qualitative assessments of surface corrosion through visual examination, there is a lack of quantitative data on the spatial progression of surface corrosion [11,[18], [19], [20], [21], [22], [23], [24], [25], [26], [27], [28], [29], [30], [31]]. Furthermore, only a limited number of studies give results on the severity of localised corrosion for Mg alloys [[32], [33], [34]] following ASTM G46-94 [35], which provides specific guidelines to evaluate pitting corrosion and describes several local parameters that quantify the severity and spatial distribution of corrosion features, including pit size, pit depth, pit density and pitting factor.

To date, few in-vivo and in-vitro studies [19,20,36,37] have quantified the non-uniform relationship between specimen corrosion and mechanical strength of magnesium alloys undergoing corrosion. While the disproportionate reduction in load-bearing capacity, compared to corresponding mass loss, has clearly attributed to pitting corrosion observed across specimens [19,20,37], these studies have provided little quantitative understanding on how pit formation (e.g. severity and spatial distribution) affects overall mechanical performance. Recently, we have established an automated detection framework that enables a fully systematic evaluation of surface corrosion through a micro-CT based detection algorithm (*PitScan*) [33]. This study identified a clearly non-linear relationship between overall mass loss and specimen strength for a magnesium-based alloy and systematically characterised the severity and spatial distribution of pitting features on the corroding surface. While this study provided important information on the relationship between spatial features of corrosion and mechanical performance, it was limited by the fact that it only considered one magnesium alloy undergoing corrosion. Of course, there is a wide range of possible alloy combinations, and the spatial and temporal progression of corrosion will likely vary extensively across these different material systems; however, it is difficult to experimentally characterise the full range of corrosion scenarios. This limits our capacity to fully understand the mechanistic relationships between surface-based corrosion and mechanical performance of these metals and alternative approaches through computational modelling are required.

Modelling approaches to predict corrosion of magnesium-based alloys are generally categorised as either physical or phenomenological approaches. While physically-based corrosion models use theoretical frameworks that capture the chemical processes taking place on the corroding surface, they are computationally prohibitive and their implementation in the finite element method generally employ moving-mesh approaches, which tend to only allow uniform corrosion, thereby limiting their ability to predict localised, non-uniform corrosion [[38], [39], [40]]. On the other hand, a wide range of phenomenological corrosion models have been proposed for magnesium-based alloys that use combinations of a continuum-based damage mechanics and/or element removal on the corroding surface to simulate mass loss. While several of these approaches have also been limited to uniform corrosion [41,42], many other models have used random distribution functions to prescribe weighted probabilities across the corroding surface that enable localised pits to form and evolve [19,20,[43], [44], [45]]. These have been shown to be superior to uniform-based models in capturing the non-linear reductions in specimen strength during corrosion [19,42,44]. However, while these models have captured non-linear reductions in strength, very few models have been directly compared to experimental samples undergoing corrosion. Therefore, it is not clear whether these models actually capture (i) the overall stress-strain behaviour of samples undergoing corrosion and (ii) whether they actually capture the severity and spatial distribution of pitting features on the corroding surface. To maximise the utility of these models in corrosion-based investigations, it is critical that robust validation of these models is carried out to fully understand how the spatial and temporal progression of corrosion impacts the mechanics of magnesium-based implants.

The objective of the current study is to establish the mechanistic relationship between the severity of localised corrosion and mechanical performance of magnesium-based specimens. To achieve this, a computational modelling approach was used, whereby a range of different corrosion profiles were generated through a surface-based corrosion model. Geometric features of these corrosion profiles were quantified using *PitScan* [33] and tensile failure of corroding samples was simulated through finite-element modelling. In the first instance, we evaluate the suitability of the surface-based corrosion modelling approach in capturing both the (i) geometric phenomenology of pitting corrosion and (ii) resulting mechanical response of corroded specimens, by comparing to in-vitro degraded samples [33]. Following this, we systematically investigate the relationships between key phenomenological features that describe pitting corrosion and the mechanical performance (ultimate strength, elastic modulus and strain at maximum strength) to establish new mechanistic insight into the performance of the magnesium-based materials undergoing corrosion.

## 2 Material and methods

### 2.1 Study design

To generate corroding profiles, we considered three-dimensional cylindrical geometries that had identical dimensions as the gauge sections of tensile dog-bone specimens used in our previous experimental study (gauge length of 18.95 mm and 3 mm diameter) [33]. Whereby immersion testing in c-SBF [46] was carried out over 28 days with weekly time points (37 °C, 5 %CO_2_). Following immersion, samples were cleaned in ethanol and fully dried with the degradation layer still attached on the sample's surfaces. Then all samples underwent microcomputer tomography scanning with subsequent uniaxial tensile tests [33]. A range of corrosion profiles were generated using an enhanced surface-based corrosion model that was initially developed by Grogan et al. and further developed by Quinn et al. [20,47] (described in more detail in Section 2.2). The surface geometric features of these corrosion profiles were fully quantified using the automated detection framework *PitScan*, which is an in-house developed python tool based on automated image processing with OpenCV [33,48]. By creating binarized cross-sectional images of corroding finite element geometries, *PitScan* enables quantification of key corrosion parameters (e.g. average pit depth, pit density, average radius loss, minimal cross section, etc.) that describe the spatial phenomenology of the surface profile (described in more detail in Section 2.3). An elastic-plastic constitutive material model was calibrated from the un-degraded experimental response, and the corroded samples were simulated under uniaxial tension to predict the mechanical response of samples as corrosion progressed (described in Section 2.4). Finally, correlations were established between mechanical properties and the geometrical features that describe the phenomenology of surface-based corrosion (Section 2.5).

### 2.2 Corrosion model

The corrosion model is based on the model originally developed by Grogan et al. (2011) [20], which is implemented in a finite element framework through a continuum damage mechanics (CDM) based approach [49]. This surface-based corrosion model has been widely implemented, by the authors and other groups [19,20,44,45,50]. The corrosion model assumes a scalar Damage factor (D) to initialize damage on corroding elements so that the effective stress tensor (${\tilde{\sigma }}_{ij}$) is:

(1) $${\tilde{\sigma }}_{ij}=\frac{\sigma_{ij}}{1-D}$$

First, a finite element mesh is created on the geometry with exposed elements to the environment defined as *active*. Including the recent adaptation of the model, described by Quinn et al. [47], we consider an enhanced surface-based approach to corrosion, whereby impurities within the specimen volume also direct corrosion processes resulting in more realistic pit shapes compared to the original model by Grogan et al. (2011). Firstly, random numbers ($\lambda_{e}$) are assigned to all elements (which potentially can degrade), using a Weibull curve, with the probability density function described in Eq. (2):

(2) $$f\left(x\right)=\gamma \;{\left(x\right)}^{\gamma -1}e^{-{\left(x\right)}^{\gamma }}$$

**Fig. 1:**
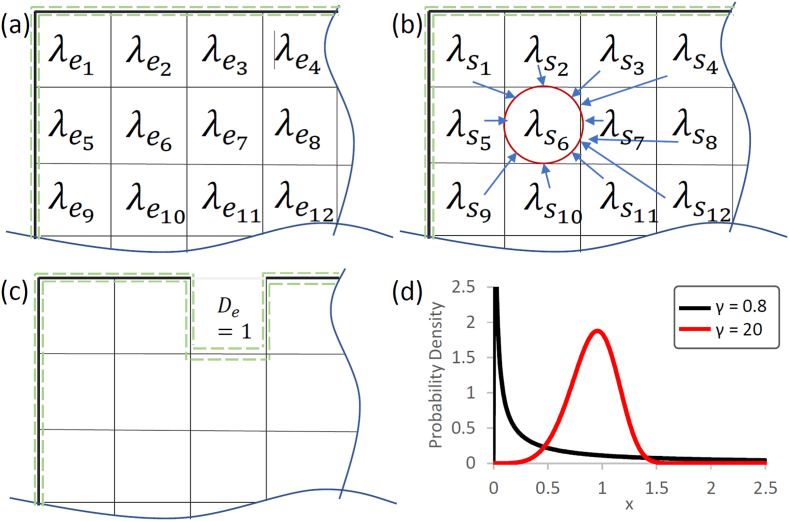
Sectional view of a finite element mesh: Basic principle of pre-processing step for the adaptation of the initial random numbers (green dashed line symbolizes the active surface) (a) initial random numbers (b) Redistribution of the random numbers, here: influence of all other elements on element 6 for the adapted random number (c) removed element where DE = 1 (d) Probability density function for a standard Weibull-Curve for pitting (γ = 0.8) and uniform corrosion (γ = 20.0).

Where γ is the dimensionless shape parameter of the probability density function with the condition x≥0 and γ≥0 (see Fig. 1 (d)), which enables modelling of localised corrosion (e.g. pitting, intergranular corrosion, etc. [8]). These pre-generated random numbers throughout the complete mesh depend only on the shape parameter γ.

The first adaptation implemented by Quinn et al. [47] uses a smoothing process to adjust the distribution, as depicted in Fig. 1(a and b). Here the influence of each element on each other is shown schematically for element number 6 (to simulate the influence of impurities/alloying elements). This smoothing process considers the random number values of all other degradable elements, including the distance from elements among themselves ($d_{ei}$) and the initial random numbers ($\lambda_{e}$):

(3) $$\lambda_{e_{max}}=max\left({\lambda_{s}}_{i}\right)=max\left({\lambda_{e}}_{i}-\left(B*d_{ei}\right)*{\lambda_{e}}_{i}\right),\;\;i=1,\;Max.\;Element\;No.$$

Where B describes the loss in pitting per unit distance, which dictates the severity of the influence of the other random numbers on each other. This step is performed once throughout the complete mesh. Then, the random numbers are normalised according to Eq. (4):

(4) $$\lambda_{e_{norm}}=\frac{\lambda_{e_{max}}}{\sum_{i=0}^{i}\lambda_{e_{max}}},\;\overset{i}{\underset{i=1}{\sum }}\lambda_{e_{norm}}=1.$$

During the final degradation step, elements that will be removed for the specified mass loss are determined by looping through all *active* elements. The current damage increment $dD_{e}$ is calculated within each *active* element according to Eq. (5):

(5) $$\frac{dD_{e}}{dt}=\;k_{u}\lambda_{e_{norm}}L_{active}$$

Where $k_{u}$ is a time dependant parameter, and $L_{active}$ is the ratio of the exposed active surface area to the respective element volume. Eq. (6) represents the addition of the increment step to the previous calculated total damage for an element:

(6) $$D_{e}=D_{e-1}+dD_{e}$$

The current total damage ($D_{e}$) of each *active* element is calculated by adding the current damage increment ($dD_{e}$) to the old total damage ($D_{e-1}$). Once $D_{e}\geq 1$ the element is removed (Fig. 1 (c)) and its adjacent elements, which were *inactive* so far, will get *active* and get included in the loop. To avoid too high single damage increments (to ensure that each element needs at least two steps to degrade), an adaption is implemented following Eq. (7):

(7) $$if\;D_{e}>D_{e_{max}},\;dt_{new}=\frac{D_{e_{max}}}{D_{e}}*dt_{old}$$

With $D_{e_{max}}=0.5$, $dt_{new}$ and $dt_{old}$ the new and old time step, respectively. For full details on the localised degradation model the authors highly recommend the original studies by Grogan et al. and Quinn et al. [20,47].

Within our study, constant values were assigned to time dependent parameters: $k_{u}=2.7\cdot 10^{8}$ to enable appropriate number of loops. In this, and previous implementations of the original corrosion model [19,20,44], executing the code directly in a VUMAT/UMAT in Abaqus requires substantial computational power, especially with the implemented adaptation from Quinn et al. [47]. To improve the efficiency of this code and enable high mesh resolution (>300,000 elements), the corrosion model was carried out as a pre-processing step through a python code, which has the additional advantage of being readily implemented in a range of finite element software codes.

By varying the shape parameter of the Weibull curve (γ) and the pitting parameter (B) of the degradation code, eight different corrosion profiles were generated from uniform corrosion to severe pitting corrosion. First, four profiles with a constant pitting parameter (B=0.8) and a varying γ (20.0,1.5,0.8,0.3) were simulated, and then γ was set constant to 0.8 and B was changed (B =1.2, 0.8, 0.5, 0.3). Additionally, γ = 0.5 and B = 0.5 was chosen as input parameter combination. Each model was degraded to 5, 10, 20, 30, 40, 50% mass loss. All models considered, had an element size of 80 μm, whereby the un-corroded geometry consisted of a total of 329,128 elements. To corrode the surface to 50% mass lass for one input set, the computational algorithm took approximately 1 h to execute on a computer workstation that had an Intel® Xeon® Gold 5118 CPU @ 2.30 GHz.

### 2.3 Automated spatial tracking of corrosion (*PitScan*)

**Fig. 2:**
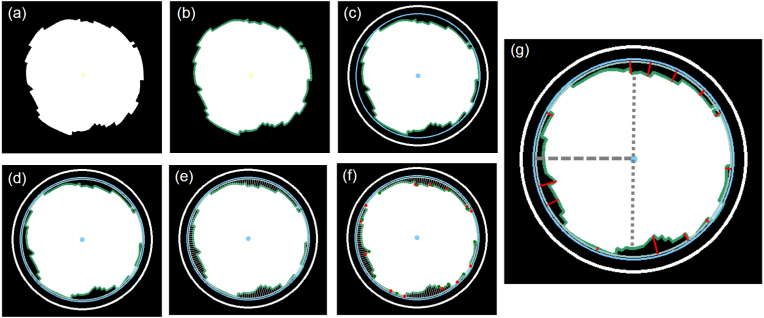
Automated image recognition process chain for FE cross sectional images (a) Raw input, (b) Contour detection, (c) Circle plotting (d) Material portion, (e) Depth tracking, (f) Pit on-off tracking, (g) Determination of deepest point of each pit. Grey dashed line fitted radius, grey dotted line minimum Mg core width, similar to van Gaalen et al. [33].

For full spatial reconstruction of the surface profile, the active finite element mesh was binarized and cross-sectional images taken every 80 μm along the y-axis (equivalent to mesh size). The approach was the same as the geometrical evaluation conducted with the μCT images from the in-vitro testing in our previous study [33]. The basic principle for the image processing for one layer is shown in Fig. 2, whereby a contour of the magnesium core and a circle fit provides the depths, in 2-degree radial increments. In the second step, the profile is reconstructed to a 3D geometry to enable all geometrical features to be quantified. A pit is defined if the detected depth exceeds 50 μm.

The *PitScan* algorithm provided quantitative information on the geometrical corrosion formation on the outer surface. Surface contour plots were generated with the tracked depth from the initial surface. Further, probability distributions were obtained and plotted that describe the frequency of pit depth ranges at the predefined mass losses. *PitScan* also evaluated key parameters that describe the spatial distribution and severity of localised corrosion. Table 1 provides a summary of these parameters and information about how they are calculated.

**Table 1:** Detailed description of generated geometrical parameters within the pit detection tool (d: single pit depth, i: number of cross-section images, r: fitted radius, r_0_ initial radius) [33].

Parameter	Symbol	Description
Pitting Factor [35]	PF	$PF=\frac{\text{deepest metal penetration }}{\text{average metal penetration}}$
Av. of ten deepest pits (μm)	${\bar{d}}_{10}$	${\bar{d}}_{10}=\frac{\sum_{0}^{x=10}d_{x}}{10}$
Pits per cm^2^	$\bar{n}$	Tracked pits per cm^2^
Volume loss through pits (%)	$VL_{pits}$	Sum of the volumes of the real pits (d > 50 μm)
Av. Radius loss	$\bar{RL}$	Average of all fitted radii for every layer: $\bar{RL}=\frac{\sum_{0}^{x=i}1-\left(r_{x}/r_{0}\right)}{i}$
Minimum fitted radius	$r_{min}$	The minimum of all fitted radii in every cross-section: $min\left(r_{x}\right)$
Minimum Mg core width	$d_{min}$	Minimum of all detected magnesium core widths: $min\left(d_{Mg}\right)$
Max. area loss in one layer	$\Delta A_{max}$	$\Delta A_{max}=max\left(1-\frac{A_{0}}{A_{x}}\right)$

### 2.4 Mechanical model

**Fig. 3:**
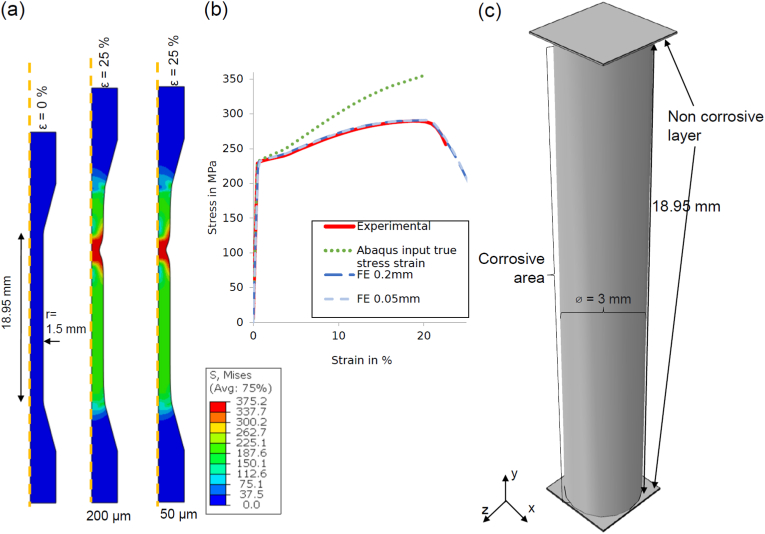
(a) axisymmetric finite element model for ε = 0% and ε = 25% for two different mesh sizes (200 and 50 μm) (b) Results calibration with axisymmetric model (c) simplified dimensions of the 3D model undergoing corrosion.

Computational modelling was carried out in the Abaqus/Explicit finite element code (DS SIMULIA, USA), with all cylindrical geometries meshed using three-dimensional reduced integration brick elements (C3D8R). Material input data for the computational model was derived based on uniaxial tensile test data measured for the WE43MEO alloy in our previous experimental study (Fig. 3 (b)). Here, the Young's modulus was E = 44.70 MPa, while a Von Mises plasticity formulation was set to define isotropic yielding [51]. The plasticity input curve was calibrated on an axisymmetric model with two different element sizes with the exact geometry of the dog bones used within the experimental study (Fig. 3 (a)) [33]. The Poisson's ratio was assumed as ν = 0.3 [52] and the density of magnesium as ρ_WE43_ = 1.84 g/cm³. Only the gauge section was used as model geometry for the final finite element analysis, to save computational time. To enable uniaxial test conditions, a layer of non-degradable elements was included at the ends of the model (see Fig. 3 (c)). Here, equational constraints were used to implement displacement-based uniaxial tension on each model. Simulations were carried out on an Intel® Xeon® Gold 5118 CPU @ 2.30 GHz with 30 CPUs and took between 1.5 h and 7 h to complete, depending on the number of remaining elements following corrosion. The effective stress-strain behaviour of corroding samples was determined based on the initial cross-sectional area of the cylindrical specimens ($A_{t_{i}}=A_{t_{0}}=\pi r²=\pi \left(1.5\;mm\right)²$). From this, the overall specimen strength was determined as the ratio of the maximum tracked Force to $A_{t_{0}}$: $\sigma_{max}=F_{max}/A_{t_{0}}$, the effective modulus was the respective elastic modulus of the linear-elastic region, while the specimen strain-to-failure was determined as the strain at. $\sigma_{max}.$

### 2.5 Data analysis

To analyse results, the key geometrical features (described in Section 2.3) were plotted against three main material properties, namely the (i) maximum specimen strength (σ_max_), (ii) effective elastic modulus (E-modulus) and (iii) strain at maximum specimen strength (ε at σ_max_). The coefficient of determination (R^2^) for a linear fitting was calculated for all data points (experimental and simulated data) to identify relationships between mechanical performance and spatial features of corrosion. In this data, results from our previous experimental study [33] characterising corrosion of Magnesium WE43MEO alloy (Meotec GmbH, Aachen, Germany) were included. This data was derived from an immersion study of cylindrical dog bone samples manufactured from a chill casted WE43MEO alloy for 28 days with weekly time steps in an incubator at 37 °C and 5% CO_2_ in simulated body fluid (c-SBF) [33,46].

## 3 Results

### 3.1 Corrosion model

**Fig. 4:**
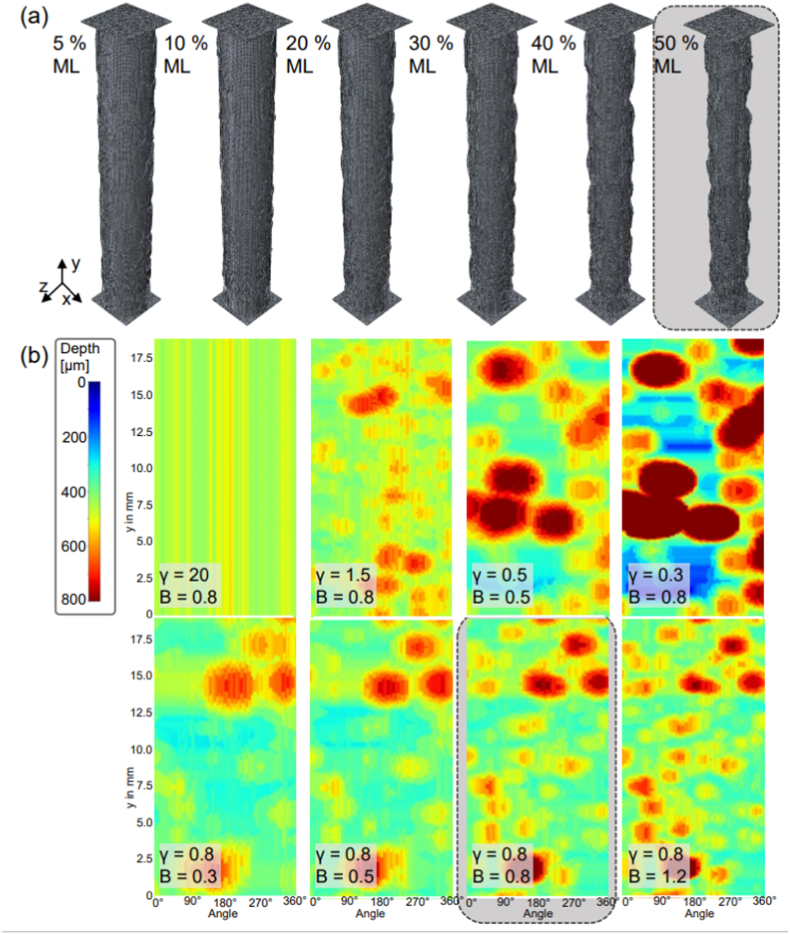
(a) Final 3D finite element models after degradation for different mass loss (ML) for one input set (γ = 0.8, B = 0.8); (b) Contour plots at 50% mass loss for the eight different corrosion scenarios of the 3D circular gauge section (r_0_ = 1.5 mm) by adapting the input values (γ,B) of the degradation model; grey highlighted figure shows the same model.

Several different corrosion profiles were generated that ranged from uniform corrosion to severe pitting/localised corrosion. Fig. 4 (a) shows resulting 3D images of the corroding cylindrical specimens for the predefined mass losses for one of the corrosion scenarios (B=0.8,γ=0.8).

Fig. 4 (b) shows 2D representations of the resulting corrosion profiles generated by *Pitscan*, whereby the depth from the original cylindrical surfaces of each specimen is plotted at 50% mass loss for each scenario. The influence of the Weibull-shape parameter (γ) on the severity of corrosion is clearly evident in the top row. Here, the spatial distribution of corrosion is almost uniform for the highest Weibull-shape parameter (γ=20), while severe pitting and highly localised non-uniform corrosion becomes evident as the Weibull-shape parameter is decreased. Meanwhile, variation of the pit parameter B dictates the pit depth, pit area and pit density (see second row Fig. 4 (b)). Higher values of B result in increasing pit density and pit depth, while the opening area of individual pits reduces. Consequently, lower values of B are attributed with wider but less deep pits and a lower density. Though, γ is still the decisive parameter which controls the severity of localised corrosion effects due to its influence on the initial random numbers.

**Fig. 5:**
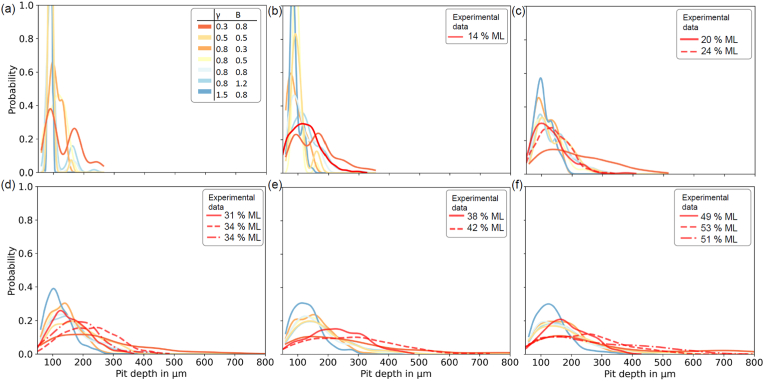
Pit depth distribution for seven localised corrosion profiles including similar experimental examined mass losses (a) 5% (no similar experimental sample available), (b) 10%, (c) 20%. (d) 30%, (e) 40%. (f) 50% Mass loss. (Legend for all given in (a)).

Fig. 5 shows the probability density functions that quantify the detected pit depths for all scenarios. Here, the experimental data from our previous study was also included (red lines, where suitable mass losses were available) [33]. In the early stages of corrosion, the distributions for all corrosion scenarios are tight as small single pits develop. Over time, these pits evolve and coalesce to form larger pits that results in wider distribution functions. Interestingly, the experimental data showed close correlation to several of the simulated pitting profiles, in particular for the scenario that had parameters of γ=0.8 and B=0.8 or B=0.5.

**Fig. 6:**
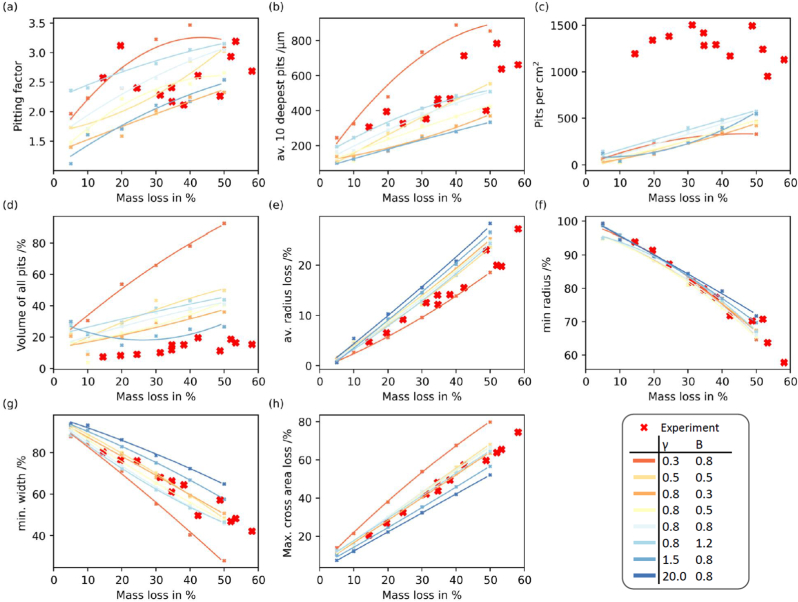
Most relevant phenomenological corrosion features vs. detected mass loss. (Plots with features related to localised corrosion are plotted without the uniform corrosion profiles (a–d)).

*PitScan* was used to quantify pitting parameters across all scenarios. Fig. 6 shows this quantitative information, whereby various pitting parameters are plotted as a function of mass loss. These curves show the evolution of pitting features as corrosion progressed for each simulated corrosion scenario. It must be noted that features relating to the evolution of pits (Fig. 6(a–d)) cannot be calculated for the uniform corrosion model (γ=20,B=0.8). This quantitative information shows the evolution of key pitting parameters is in many cases to the non-uniformity of prescribed corrosion model parameters. For example, features such as pitting factor (Fig. 6 (a)), average of 10 deepest pits (Fig. 6 (b)), volume of all pits (Fig. 6 (d)), show largest increases for (γ=0.3,B=0.8), with these changes becoming more modest as the corrosion scenario is more uniform. This is particularly true in the case of Pitting factor [35], whereby values close to 1 depict more uniform formation, while higher values are attributed to more pitted profiles (Fig. 6 (a)). Profiles with the same initial shape parameter of the Weibull-curve (γ=0.8) resulted in similar trends in average radius loss (Fig. 6 (e)), minimum width (Fig. 6 (g)) and maximal detected area loss (Fig. 6 (h)) across all pit parameter (B) values. For almost all parameters, the experimental data is distributed throughout the simulated corrosion scenarios indicating that the corrosion model is quite effective in replicating the spatial phenomenology of surface-based corrosion. The only exception here is the corrosion model's capacity to predict the pit density (see Fig. 6 (c)), which is limited due to the finite mesh dimensions (80 μm).

### 3.2 Mechanical modelling

#### 3.2.1 Model calibration

Model parameter fitting was carried out by calibrating the mechanical model to experimental data through an axisymmetric model of the tensile dog-bone specimens and the results of this process are shown in Fig. 3 (b). This model accurately captures key features of the nominal stress/strain response determined from experiments, with the hardening and ultimate tensile strength of the magnesium correctly predicted by the model. Furthermore, the contour plots of these simulations (Fig. 3 (a)) demonstrate that the ultimate tensile strength is reached due the predicted necking behaviour of the ductile Magnesium alloy, which was also observed in experiments. The Considère criterion says that necking occurs when true stress reaches the strain hardening rate [53], whereby $\frac{d\sigma_{true}}{d\varepsilon_{true}}=\sigma_{true}$. Prior to necking the effect of strain-hardening is stronger than the effect of the area reduction. With an increase of strain this phenomenon reverses, which leads to the formation of necking. This phenomenon in perfect models was also reported by Joun et al. in detail [54]. The behaviour was demonstrated to be independent of mesh sensitivity effects, whereby both 200 and 50 μm element size showed similar responses (see Fig. 3 (b)).

**Fig. 7:**
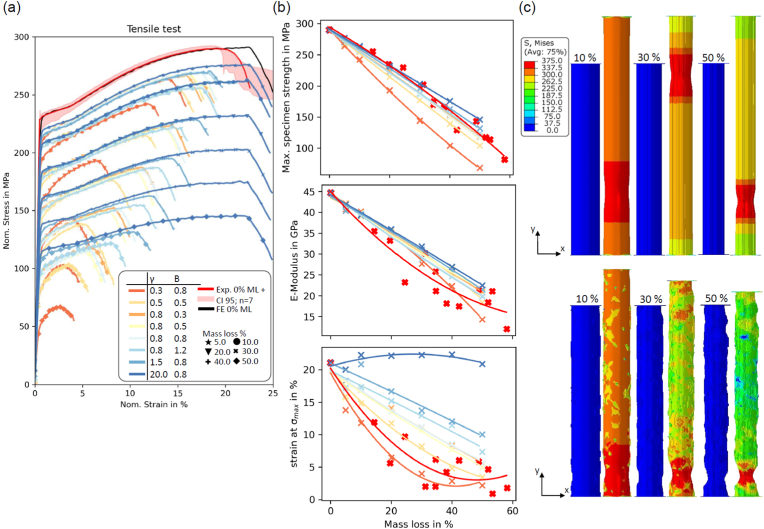
(a) Uniaxial tensile tests: experimental data (red area: Confidence Interval (CI) for n = 7 of undegraded samples) vs. simulated data (including data for several mass losses and the eight pit scenarios); (b) Maximum specimen strength (σ_max_), Elongation at σ_max_ and effective E-Modulus over Mass loss for the eight corrosion scenarios and the experimental data set; lines are second degree polynomial fits for each scenario; (c) contour plots showing Von Mises stress distribution for two different model input parameters (on top: γ = 20, B = 0.8; bottom: γ = 0.8, B = 0.8) at three different mass losses each unloaded and maximal loaded under uniaxial tension.

Fig. 7 (a) shows the results of the calibrated model, which has been extended to three dimensions. For the uncorroded sample (0% mass loss) there is only a slightly higher elongation compared to the axisymmetric model used for the fitting process, with the stress-strain response of the WE43MEO alloy still fully captured.

#### 3.2.2 Mechanical performance of corroding samples

Fig. 7 (a) shows the simulated uniaxial tensile response for the corroding samples of all considered scenarios. In general, the specimen strength and elongation decrease for higher mass loss percentages. However, for the uniform model (γ=20.0,B=0.8), there is no reduction in the strain at σ_max_ as corrosion progressed. Fig. 7 (b) shows that there is a strong relationship between specimen strength (σ_max_) and mass loss for all computational scenarios considered, with many of these capturing the trend observed experimentally. σ_max_ is mostly taken as the relevant factor for calibrating a degradation model to an experimental data set [19,20]. The relationship between strain at σ_max_ (Fig. 7 (b) bottom) shows greater variation in the predicted results across different corrosion scenarios. This clearly demonstrates that uniform corrosion is not suitable as a corrosion model for the examined WE43MEO alloy, as it does not capture any reduction in strain at σ_max_. The effective E-Modulus over mass loss (Fig. 7 (b) middle) follows largely similar behaviour to the trends seen for specimen strength (σ_max_). Overall, the severely pitting models (γ=0.3,B=0.8 or 0.5) showed the best agreement with the properties determined experimentally. Contour plots of the Von Mises stress are presented in Fig. 7 (c) for the unloaded and maximal loaded step, respectively. Exemplarily the uniform model and a pitted profile is taken with different mass losses. All models show the necking behaviour which is also observed within the tested dog bones.

### 3.3 Correlations

**Fig. 8:**
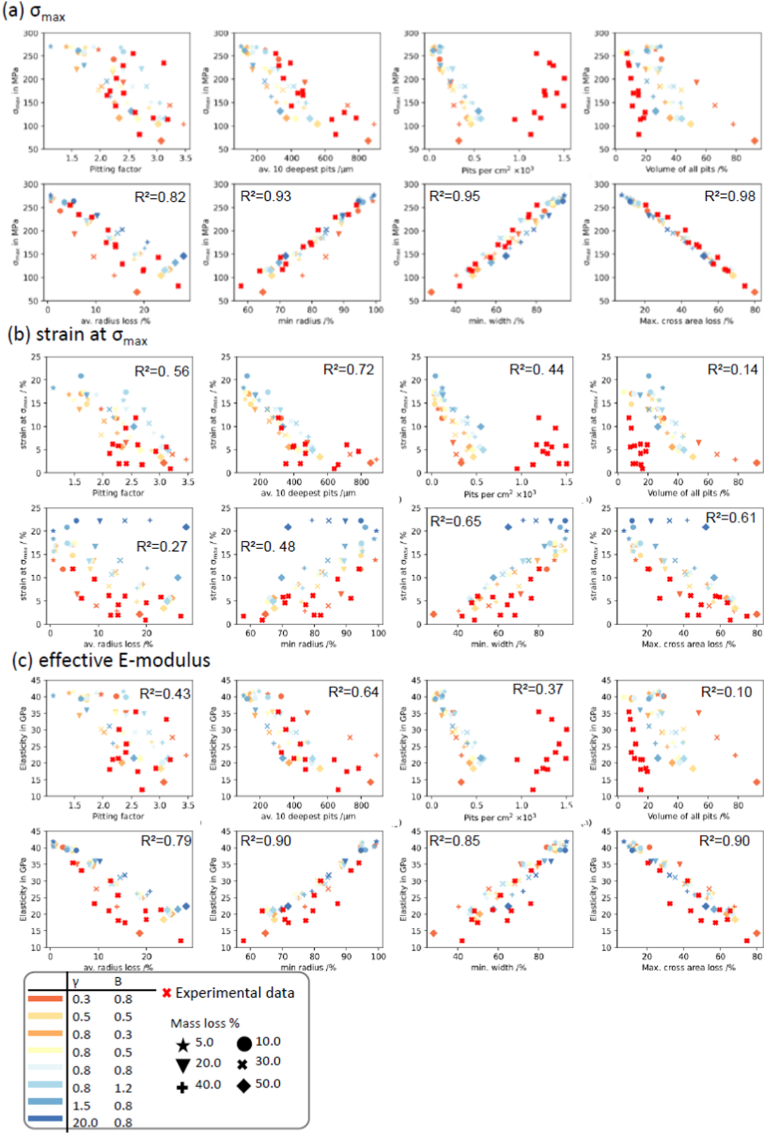
Correlation plots of eight most important phenomenological corrosion features over (a) remaining specimen strength σ_max_, (b) strain at σ_max_, (c) effective E-modulus. First row each, are features belonging to the formation of pits so the uniform model (γ = 20.0) was excluded.

Having proved the suitability of the corrosion model in capturing the phenomenology of corrosion (section 3.1), as well as the mechanical mechanistic (section 3.2), we can establish relations between key pitting parameters and the mechanical integrity. Fig. 8(a–c) shows correlation plots that established quantitative relationships between the pitting parameters calculated by *PitScan* and the predicted mechanical parameters (σ_max_, strain at σ_max_, and the effective E-Modulus). Also, included here are results from our previous experimental study. Again, it must be noted that the uniform model (γ=20.0,B=0.8) was excluded for features which are related to the formation of pits (e.g. pit depth, pit density, pitting factor, etc.). In the supplementary data a similar figure is shown with only the evaluation with a uniform model and a localised corrosion model. The parameters described in ASTM G46-94 [35] for evaluating pitting corrosion were taken as a starting point for this analysis and extended by several more suitable features, which potentially are linked to the mechanical integrity. A coefficient of determination was calculated for all plots by consider all data presented. Features with high R^2^ values are considered to be independent of profile formation, while features with low R^2^ values are highly dependent on the corrosion profile.

In Fig. 8 (a), there is a clear correlation between features linked to the reduction of the cross-sectional area and the specimen strength (σ_max_), with minimum radius, minimum width and max cross sectional area loss all having R-square values > 0.9. It is important to note that these pitting parameters are independently correlated to specimen strength (σ_max_) across all simulated scenarios and the available experimental data. Potential scenario-based features can be also identified in Fig. 8 like the pitting factor (R^2^ = 0.53), volume loss by pits (R^2^ = 0.27) and the average radius loss (R^2^ = 0.82). On the other hand, the detected maximal cross-sectional area loss shows an almost linear correlation to the remaining specimen strength, and no distinguishable trends for the profiles are visible. No correlation was observed for pit density, which was again likely due to the finite mesh dimensions used in the computational model (80 μm).

The strain at maximum specimen strength (Fig. 8 (b)) shows for all features distinguishable trends between the tested scenarios. Even features which are scenario independent for σ_max_ and the effective E-modulus are scenario-dependent for the maximum strain (like minimum width, max. cross-sectional area loss, minimum fitted radius). This behaviour underlines the importance of considering the strain value for calibrating degradation models. The manner of the formation of pits seems to highly dictate the strain response, and a uniform model is not suitable as a degradation model, because of the non-reduction in strain over several mass losses Fig. 8 (c) shows the relationship between the pitting features and the effective E-modulus. In general, a similar response compared to σ_max_ is visible, however the dependence of effective E-modulus on the maximal cross-sectional area loss is not as clear as was observed for σ_max_.

## 4 Discussion

In this study, a computational framework was developed to establish mechanistic relationships between the localised corrosion and mechanical performance of a magnesium-based alloy. A finite element-based corrosion model [20] was used to generate corrosion profiles, with subsequent uniaxial tensile test simulations to track the mechanical integrity. The python-based detection framework *PitScan* provide detailed quantification of the phenomenological features of corrosion, including a full spatial tracking of surface-based corrosion. Through this approach, this study is the first to quantitatively demonstrate that a surface-based non-uniform corrosion model can capture both the geometrical and mechanical features of a magnesium alloy undergoing corrosion by comparing to experimental data [33]. Using this verified corrosion modelling approach, this study evaluated a wide range of corrosion scenarios and enabled quantitative relationships to be established between the mechanical integrity and key phenomenological corrosion features. In particular, we demonstrated that parameters that were directly linked to the reduction of the cross-sectional area of the specimen were the best predictors of mechanical performance.

Rapid mechanical deterioration of magnesium-based medical implants has limited their implementation in load-bearing applications [55,56]. While the accelerated loss of mechanical integrity of specimens undergoing corrosion has previously been linked to pitting corrosion through qualitative means [19,20], there has been a lack of understanding mechanistic relationships between corrosion and mechanical performance. Here, we clearly demonstrate that the deterioration in mechanical performance of corroding specimens is directly linked to localised corrosion and we provide a comprehensive set of quantitative relationships between surface-based pit formation and the mechanical performance, described by specimen strength (σ_max_), strain at maximum strength (ε at σ_max_) and Young's modulus (E). Here, our data set shows that the minimal cross-sectional area is the strongest predictor of the remaining mechanical strength (R^2^ = 0.98), and this parameter is independent of the severity or spatial features of localised surface corrosion. Interestingly however, minimal cross-sectional area was not predictive of the failure strain of the specimen, whereby there was substantial variation of this parameter with the various profiles simulated, particularly when uniform corrosion scenarios were considered. Instead, it was found that parameters relating to the deepest pits better correlated with strain at failure. From experimental data, there is a clear reduction of the strain-to-failure in corroding specimens as [19,20,33,37], which is likely a result of localised corrosion providing suitable imperfection(s) that allow damage to localise. On the other hand, minimal cross-sectional area did show good correlation with the reducing remaining elastic modulus (R^2^ = 0.98), which was again independent of the corroding profile considered. Interestingly, our analysis demonstrated that parameters described in ASTM G46-94 (e.g. pitting factor, average of ten deepest pits etc.) showed weaker correlations to the mechanical integrity of corroding specimens, highlighting the importance of considering the other parameters highlighted here (e.g. minimum cross sectional area).

This study quantitatively demonstrates that a finite element-based corrosion model can capture both the geometrical and mechanical features of a magnesium alloy undergoing corrosion. Until now, many other corrosion-based models have assumed uniform corrosion [[38], [39], [40], [41], [42]], or have implemented non-uniform surface-based corrosion through stochastic approaches, but have not presented any validation of approaches in capturing geometric features of corrosion, or associated implications on mechanical performance. Instead, there has tended to be calibration of model parameters to fit the bulk mechanical performance over time [19,20,43,44]. Our study has shown that it is possible to control the geometrical formation of localised corrosion features by two factors (γ and B) of the corrosion model. We believe with that this corrosion model framework can be used for any metal type that undergoes surface-based corrosion, and is not limited to magnesium-based alloys. Furthermore, the corrosion model presented here not only enables control of the severity of pitting, but it is also possible to adjust the width, depth and density of pits evolving. Plotting corrosion features over the predefined mass loss resulted in clear trends for each profile (Fig. 6), so that each set is clearly distinguishable from each other. Pitting factor described in ASTMG46-94 [35] follows this trend and our calculated values show that a pitting factor of 1 describes a uniform behaviour, while higher values are related to more pitted profiles. We observe an increasing pitting factor with an increase of mass loss in all scenarios considered. Importantly, our findings demonstrated that a uniform degradation model is not suitable for simulating the mechanical response for Magnesium alloys that are undergoing corrosion, i.e., degrading in the human body. Uniform corrosion models have two major failings in that the predicted reduction in specimen strength is proportional to the mass loss and the strain at failure remains largely constant for all simulated mass losses (see Fig. 8). Although it was not the initial goal of our study, we also identified a combination of γ and B, which tend to fit best the experimental data in terms of both geometric and mechanical performance.

In addressing the limitations of the current study, it should be noted that the corrosion model fails to replicate the pit density that was observed experimentally, which is a direct result of the limited element size within the finite element mesh. However, this is necessary to achieve simulated results within an appropriate computational time. These plots clearly show the limits of the finite element model in predicting the pit density (Pits per cm^2^). Here all models weaken to replicate the experimental data due to the limits of the mesh size. However, it was found experimentally that this feature does not have a strong correlation with mechanical parameters and, instead, the corrosion features that dominate the mechanical response are related to larger scale parameters (e.g. minimum radius), which are represented quite well in the model meaning that the mechanical response can still be captured. Additionally, the used phenomenological degradation model neglects the formation of the outer degradation layer with its ion compositions. However, to include its effect on the overall degradation with a sufficient mesh sensitive with subsequent uniaxial tensile test requires a massive computational power. Further, we want to mention that stress corrosion cracking effects where fully neglected, which could accelerate degradation [20,57,58]. Further it must be noted, that in-vitro tests were used as a benchmark, however the corrosion mechanism in the human body can vary a lot. Nevertheless, the focus of the current study is on the correlation between any pit formation and the relating mechanical response, independently how and where it evolves. Often lower degradation rates in-vivo were reported than in in-vitro tests (TRIS- or HEPES-buffered medias) [59,60]. Here, an accelerated degradation rate is favourable to achieve reasonable mass losses within an appropriate time period.

## 5 Conclusions

This study demonstrated that a surface-based non-uniform corrosion model can capture both the geometrical and mechanical features of a magnesium alloy undergoing corrosion by comparing to experimental data. Using this verified corrosion modelling approach, this study demonstrated that the minimal cross-sectional area parameter was the strongest predictor of the remaining mechanical strength (R^2^ = 0.98), with this relationship being independent of the severity or spatial features of localised surface corrosion. Our findings demonstrated that a uniform degradation model is not suitable for simulating the mechanical response for Magnesium alloys that are undergoing corrosion. Interestingly, our analysis demonstrated that parameters described in ASTM G46-94 showed weaker correlations to the mechanical integrity of corroding specimens, compared to parameters determined by *Pitscan*. This study establishes new mechanistic insight into the performance of the magnesium-based materials undergoing corrosion within biological environments.

## Ethics approval and consent to participate

This publication does not consider any animal-, human-, in-vivo experimental data, so no ethical approval was necessary.

## CRediT authorship contribution statement

**Kerstin van Gaalen:** Methodology, Investigation, Software, Writing – original draft, Visualization. **Conall Quinn:** Methodology, Conceptualization. **Felix Benn:** Investigation, Conceptualization, Writing – review & editing, review. **Peter E. McHugh:** Conceptualization, Writing – review & editing, review. **Alexander Kopp:** Conceptualization, Writing – review & editing, review, Supervision. **Ted J. Vaughan:** Conceptualization, Writing – review & editing, Funding acquisition, Supervision.

## Declaration of competing interest

The authors declare that they have no known competing financial interests or personal relationships that could have appeared to influence the work reported in this paper.
