# Supplementary material for: Linking the effect of localised pitting corrosion with mechanical integrity of a rare earth magnesium alloy for implant use
Source: Bioact Mater. 2022 Aug 12;21:32–43. doi: 10.1016/j.bioactmat.2022.08.004 (PMC9396051; doi:10.1016/j.bioactmat.2022.08.004)
Supplement: Multimedia component 1 [file mmc1.pdf]

Linking the Effect of Localised Pitting Corrosion with Mechanical Integrity of a Rare Earth Magnesium Alloy for Implant use

Kerstin van Gaalen, Conall Quinn, Felix Benn, Peter E. McHugh, Alexander Kopp, Ted J. Vaughan

Supplementary data

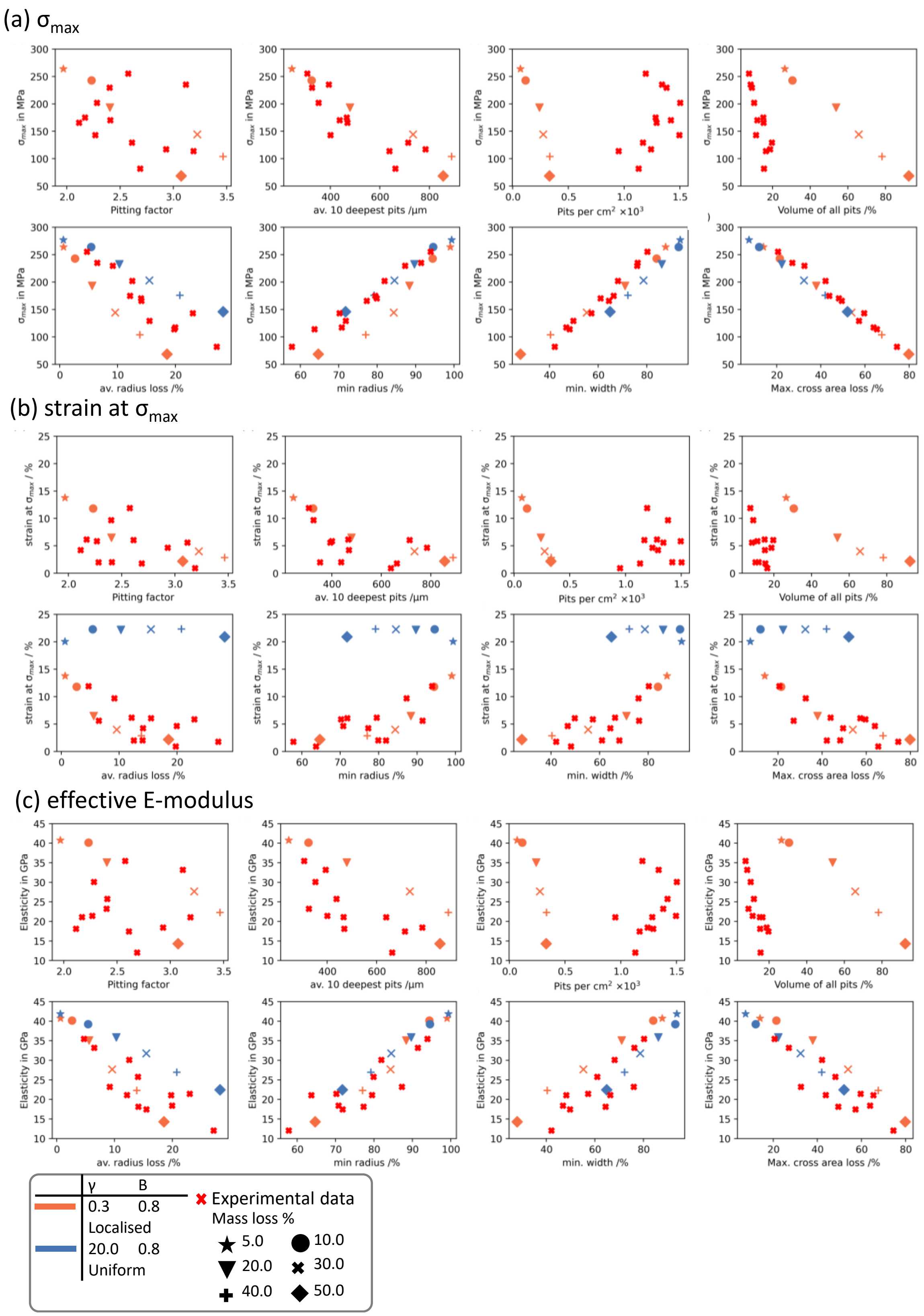

Figure S1: Correlation plots of eight most important phenomenological corrosion features over (a) remaining specimen strength σ_{max}, (b) strain at σ_{max}, (c) effective E-modulus. First row each, are features belonging to the formation of pits so the uniform model (γ=20.0) was excluded.
